# Reliable *in vitro* method for the evaluation of the primary stability and load transfer of transfemoral prostheses for osseointegrated implantation

**DOI:** 10.3389/fbioe.2024.1360208

**Published:** 2024-03-21

**Authors:** Giulia Galteri, Marco Palanca, Domenico Alesi, Stefano Zaffagnini, Kavin Morellato, Emanuele Gruppioni, Luca Cristofolini

**Affiliations:** ^1^ Department of Industrial Engineering, School of Engineering and Architecture, Alma Mater Studiorum-University of Bologna, Bologna, Italy; ^2^ IRCCS Istituto Ortopedico Rizzoli, Bologna, Italy; ^3^ Centro Protesi INAIL, Bologna, Italy

**Keywords:** lower-limb amputation, above-the-knee amputation, transfemoral osseointegrated prosthesis, primary stability, load transfer, *in vitro* mechanical test, micromotion

## Abstract

Osseointegrated transfemoral prostheses experience aseptic complications with an incidence between 3% and 30%. The main aseptic risks are implant loosening, adverse bone remodeling, and post-operative periprosthetic fractures. Implant loosening can either be due to a lack of initial (primary) stability of the implant, which hinders bone ingrowth and therefore prevents secondary stability, or, in the long-term, to the progressive resorption of the periprosthetic bone. Post-operative periprosthetic fractures are most often caused by stress concentrations. A method to simultaneously evaluate the primary stability and the load transfer is currently missing. Furthermore, the measurement errors are seldom reported in the literature. In this study a method to reliably quantify the bone implant interaction of osseointegrated transfemoral prostheses in terms of primary stability and load transfer was developed, and its precision was quantified. Micromotions between the prosthesis and the host bone and the strains on the cortical bone were measured on five human cadaveric femurs with a typical commercial osseointegrated implant. To detect the primary stability of the implant and the load transfer, cyclic loads were applied, simulating the peak load during gait. Digital Image Correlation was used to measure displacements and bone strains simultaneously throughout the test. Permanent migrations and inducible micromotions were measured (three translations and three rotations), while, on the same specimen, the full-field strain distribution on the bone surface was measured. The repeatability tests showed that the devised method had an intra-specimen variability smaller than 6 μm for the translation, 0.02 degrees for the rotations, and smaller than 60 microstrain for the strain distribution. The inter-specimen variability was larger than the intra-specimen variability due to the natural differences between femurs. Altogether, the measurement uncertainties (intrinsic measurement errors, intra-specimen repeatability and inter-specimen variability) were smaller than critical levels of biomarkers for adverse remodelling and aseptic loosening, thus allowing to discriminate between stable and unstable implants, and to detect critical strain magnitudes in the host bone. In conclusion, this work showed that it is possible to measure the primary stability and the load transfer of an osseointegrated transfemoral prosthesis in a reliable way using a combination of mechanical testing and DIC.

## 1 Introduction

Lower limb amputation has a significant effect on the patients’ quality of life. According to the World Health Organization, the global population of people with amputation is about 40 million (Marino et al., 2015; Windrich et al., 2016) and can only be expected to increase (Ziegler-Graham et al., 2008).

Currently, the connection to the residual limb through a socket represents the *gold standard* technique used on transfemoral amputation (Gerzina et al., 2019). However, sockets induce skin lesions in more than 50% of transfemoral amputees, resulting in a negative impact on mobility and mechanical stability, and in a high abandon rate of use of transfemoral prostheses (25%–57%) (Hagberg and Brånemark, 2001; Paternò et al., 2018). An alternative solution is being offered by osseointegrated prostheses, which reported a high satisfaction rate of patients (Örgel et al., 2022). These prostheses provide a direct structural connection between the external prostheses and the remaining living bone showing associated advantages, such as enhanced proprioception (often referred to as osseoperception) and a better range of motion (ROM) at the hip (Hebert et al., 2017; Hagberg et al., 2008). Mechanical stability of osseointegrated implants is granted by the ingrowth of bone tissue in the porosities of the implant surface. Despite the clinical advantages of the osseointegrated transfemoral prostheses, failure does occur due to septic or aseptic causes with an incidence between 3% and 30% (Juhnke et al., 2015; Aschoff and Juhnke, 2016; Hagberg et al., 2020; Muderis and Glatt, 2016; Leijendekkers et al., 2017; Atallah et al., 2018; Hansen et al., 2019; Ranker et al., 2020; Reetz et al., 2020; Örgel et al., 2020). Septic complications mainly derive from soft tissue infection at the stoma. The aseptic reasons for revision are mainly post-operative implant loosening, and periprosthetic fractures. To reduce the risks of loosening, the implantation of osseointegrated prostheses is commonly performed in two stages. This allows the bone ingrowth before load bearing (unlike common practice in total hip and total knee replacement). Implant loosening can either be due to a lack of initial (primary) stability of the implant, which hinders bone ingrowth and therefore prevents secondary stability, or, in the long-term, to the progressive resorption of the periprosthetic bone. Indeed, the alterations of the bone loading due to the use of stiff implants induce bone loss, contributing to the loss of mechanical stability. Post-operative periprosthetic fractures are most often caused by stress concentration (Juhnke et al., 2015; Örgel et al., 2021). Other mechanical complications, such as mechanical failure of the intramedullary stem or of the abutment or of the dual cone are more likely to occur in the long-term, especially among the more physically active patients (Hagberg and Branemark, 2001; Hagberg et al., 2020).

Primary stability has been widely assessed in hip prosthesis through both *in vitro* test and *in silico* models evaluating the permanent migrations and the inducible micromotions (Monti et al., 1999; Viceconti et al., 2006; Østbyhaug et al., 2010). Only few previous experimental *in vitro* studies, instead, focused on the primary stability of the osseointegrated transfemoral implant in terms of inducible micromotions (Barnes et al., 2019) and strain distribution (Cristofolini et al., 2009; Tomaszewski et al., 2013; Ahmed et al., 2020). The methods published allow the measurement either of the implant micromotions or of the strain distribution separately, but do not allow to assess both micromotions and strain distribution simultaneously, on the same specimen. *In silico* models were used to assess the primary stability and/or the strain distribution around the osseointegrated transfemoral prosthesis (Xu et al., 2006; Tomaszewski et al., 2010; Tomaszewski et al. 2012; Tomaszewski et al. 2012; Newcombe et al., 2013; Prochor and Sajewicz, 2018; Prochor and Anna Mierzejewska, 2019; Ahmed et al., 2020). However, the only validated model for the evaluation of the load transfer was proposed by Ahmed and by Tomaszewski (Tomaszewski et al., 2013; Ahmed et al., 2020).

The methods to experimentally investigate the primary stability and load transfer are far from consolidated. Indeed, quite different methods, criteria and metrics are reported in the literature (Galteri and Cristofolini, 2023). Linear displacement transducers were sometimes used to measure the micromotions between the implant and the host bone, in one direction at a time. Using multiple linear transducers to measure a combination of rotations and translations is possible, but quite complex and associated with larger measurement errors. The load transfer has been assessed by measuring bone strains with strain gauges, but these can only be used on selected points. For these reasons, results from previous studies are difficult to compare or even conflicting. Moreover, the reliability and repeatability of the published methods are not always reported but are essential when these tests are used to draw decisions with a clinical impact. A protocol that simultaneously evaluates the primary stability and the load transfer and reports an analysis of the measurement errors is missing in the literature. This would improve the pre-clinical assessment of transfemoral osseointegrated prostheses. Digital Image Correlation (DIC) could be useful in overcoming these limitations. In fact, DIC is an optical technique that allows to measure full-field displacements and strain distribution (Freddi et al., 2015), and has successfully been used in biomechanics (Palanca et al., 2016).

The present study aims to define a method to quantify the bone-implant interaction of osseointegrated transfemoral prostheses. Therefore, a typical commercial osseointegrated implant was selected and used as test bench for the test protocol. In particular, the uncertainties of the method were assessed in terms of systematic and random measurement errors, intra-specimen test repeatability, and inter-specimen variability to simultaneously measure i) the primary stability of the implant, in terms of permanent migrations and inducible micromotion, and ii) the load transfer in terms of strain distribution on the host bone.

## 2 Materials and methods

To develop and tune the testing procedure, composite femur (Mod. 3406, 4th gen, Pacific Research Labs, United States of America) was firstly used, both for ethical reasons and to ensure better reliability and repeatability of the experiment (details are not presented here for brevity). Then, in order to test the applicability and evaluate the measurement errors, cadaveric human femurs were used. Digital Image Correlation (DIC) was used to measure the relative displacements between the implant and the bone, and the strain distribution on the bone surface. The measurement errors, the intra-specimen repeatability and inter-specimen variability affecting the displacements and the strains were quantified.

### 2.1 Specimens preparation

The study complied with the Declaration of Helsinki and was approved by the Ethical Committee of the University of Bologna (reference n. 113063, 10^th^ May 2021). Five human cadaveric femurs from four male and one female donors (52 ± 7 years old, Table 1) were obtained through an ethically approved international donation program (Anatomy Gift Registry, United States). The specimens were kept hydrated and stored at −28°C to avoid alteration of the mechanical properties.

**TABLE 1:** Donors details and size of the stem implanted.

Specimen ID	Age (year)	Sex	Right/Left	BMI (kg/m^2^)	Implant size
1	58	male	L	34	15
2	40	male	L	32	17
3	52	male	R	24	15
4	52	female	R	22	17
5	56	male	R	35	16
Mean ± SD	52 ± 7			29 ± 6	

**FIGURE 1:**
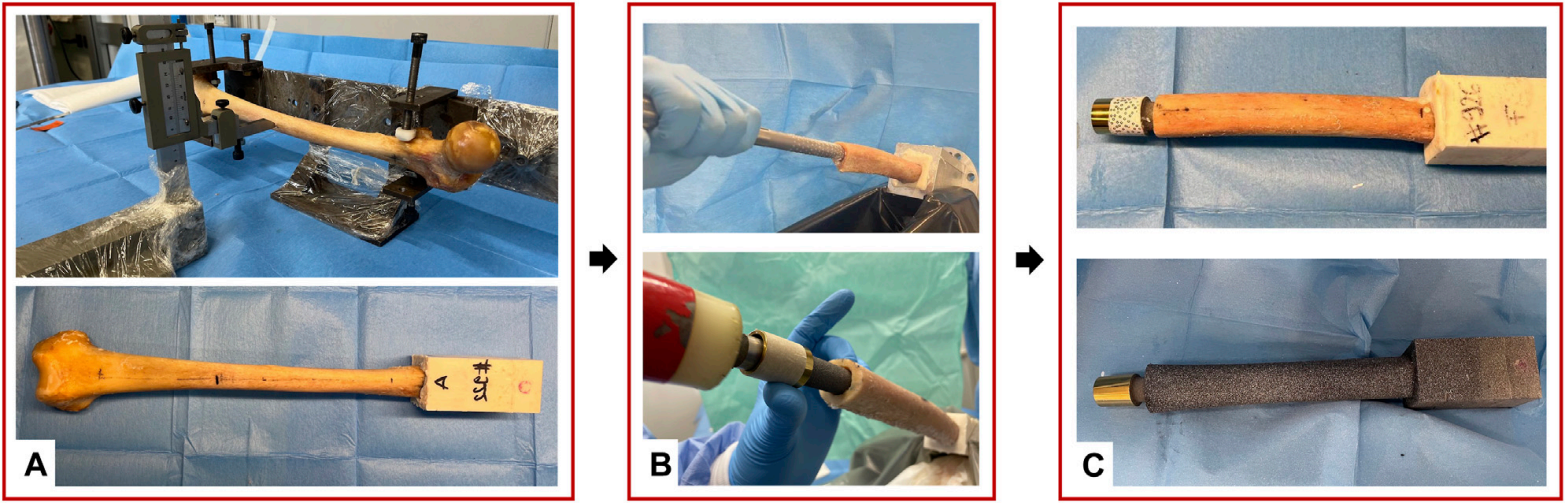
Overview of the preparation of the specimens: **(A)** standardized alignment based on anatomical landmarks and potting of the proximal part of the specimens; **(B)** Reaming and rasping of the femoral canal and the press-fit implantation; **(C)** Speckle pattern preparation with the white on black water-based paint; the distal extremity of the prosthesis was equipped with a set of markers.

Each specimen was scanned with a computed tomography (CT, VCT LightSpeed, GE medical systems, United States, slice thickness = 0.6 mm, in-plane resolution = 0.5 mm) to determine the anatomy of the bone, the dimension of the femoral canal, as well as to allow the selection of the implant size. The soft tissues were carefully removed using surgical tools. The specimens were aligned according to a procedure of “*Anatomical Reference frames for long bones: Biomechanical Application*” from (Preedy, 2012). For each specimen, the femoral head was cut, and the proximal part of the femur was embedded in an aluminum pot by using acrylic cement (Figure 1A). An osteotomy was performed, removing the distal 200 mm of each femur. To prepare the medullary canal, reamers were used, followed by rasps of increasing size (diameters from 15 mm to 17 mm) until the size planned based on the CT images. Then, the stem was press-fitted into the bone (Figure 1B).

### 2.2 Prosthetic implant

**FIGURE 2:**
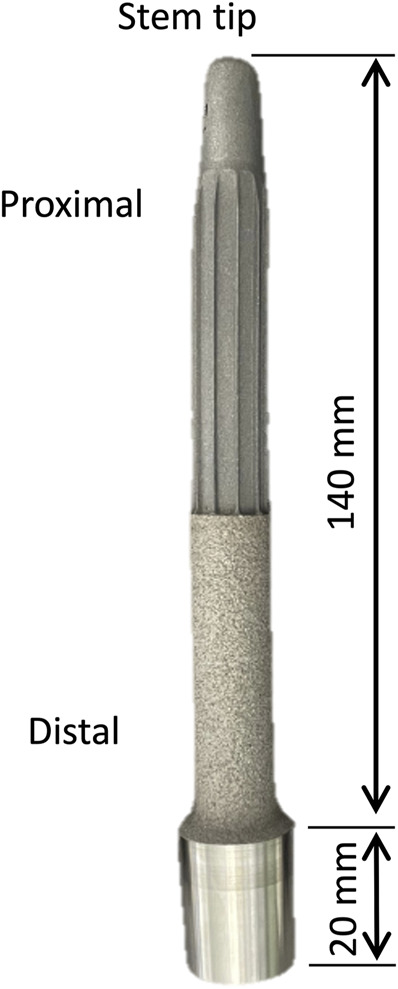
Replica of the Badal X stem used in the experimental mechanical tests. The main dimensions of the stem are indicated.

Replicas of the Badal X stem (OTN Implants) were generated with a reverse engineering on CAD (Inventor, Autodesk, United Stated) and 3D printed (Lincotek, Italy) from pure titanium (Elastic modulus = 110 GPa) (Figure 2). The Badal X implant has a constant curved stem to fit the physiological curve of the femoral canal. The distal section has a plasma-sprayed rough titanium coating to improve the osseointegration. Three different sizes of stem were implanted (Table 1).

### 2.3 Loading protocol and loading set-up

To evaluate the primary stability and load transfer in a relevant loading condition, the testing protocol was devised to simulate level walking, which is the most frequent motor task, especially in the early post-operative period. Published datasets providing loads measured during daily life activities (Dumas et al., 2017; Lee et al., 2007; Bergmann et al., 2014; Pather et al., 2018) were examined to identify the relevant values of forces and moments. In particular, the testing protocol focused on the load peak occurring during gait: according to (Frossard et al., 2021), the largest moment occurs around the mediolateral axis at the heel strike (20–40 Nm).

In order to test this relevant condition, the femur was tilted by 10° in flexion and 10° in adduction. To mimic the length of the prosthesis’s external components from the osteotomy down to the knee, a metallic prosthesis extension of 200 mm was used. The metallic prosthesis extension was rigidly connected to the prosthesis through an M14 thread.

**FIGURE 3:**
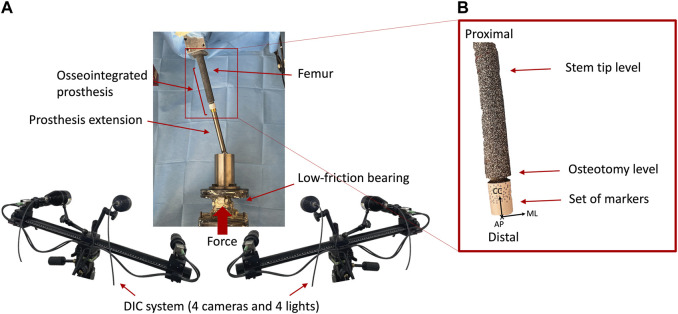
**(A)** Overview of the Experimental setup of the mechanical test: a uniaxial-servo-hydraulic testing machine and a dedicated system of low-friction linear bearing were used to deliver the force to the distal end of the specimen, while the proximal extremity was fully constrained. The prosthesis extension was used to mimic the prosthesis’ external components down to the knee and was connected to the osseointegrated prosthesis. The four cameras of the DIC viewed the implanted specimen from a mediolateral and anterior view. **(B)** Zoomed detail of the tested specimen. The reference system adopted is indicated, where the axes were aligned in medio-lateral (ML), cranio-caudal (CC), and antero-posterior (AP) directions.

A uniaxial-servo-hydraulic testing machine (8500 Instron, UK) equipped with a 10 kN load cell was used to apply the load. The specimens were fully constrained proximally, using the acrylic cement pot, and a force was applied distally resulting in a combination of compression and bending moment. To avoid transmission of any undesired component of load, free horizontal translations were granted by means of low-friction linear bearings (Figure 3). A preload of 150 N was applied. Then, each specimen was loaded by applying 100 sinusoidal cycles in loading control: load between 150 N and 850 N, at 1 Hz resulting in a combination of compression and bending moment of 30 Nm at the stem tip level.

### 2.4 Preparation of the speckle pattern and of the markers for DIC acquisition

A high-contrast white-on-black speckle pattern was prepared on the bone surface before mechanical testing (Figure 1C). A uniform background was first prepared spraying a matt black water-based paint (Nero Opaco, Axerons, Italy). The speckle pattern was created using matt white water-based paint (Q250201 Bianco Opaco, Chreon, Italy) thinned with 40% of water and sprayed using an airbrush air gun (nozzle 1.8 mm). The distance between the specimens and the airbrush air gun was set to 1,000 mm, while the pressure was set to 1,000 kPa in order to obtain the desired dot size, following an approach similar to (Lionello and Cristofolini, 2014). More details about the distribution of dimensions of the speckle dots can be found in the Supplementary Material.

Additionally, the distal portion of the prosthesis, which protruded distally to the femur osteotomy, was equipped with a set of glossy, passive circular markers (type: 0.8 mm, GOM Aramis, Braunschweig, Germany) to track the prosthesis displacements.

### 2.5 DIC acquisition

A 3D digital image correlation system (Aramis Adjustable 12M, GOM, Braunschweig, Germany) was used to measure the relative displacement between the prosthesis (fiducial markers) and the host bone (speckle pattern) and the full-field strain distribution of the femur throughout the mechanical tests. Images were acquired by four cameras (12 MegaPixels 4096 × 3000 pixels, 8 bit) equipped with high quality 75 mm lenses (f 4.5, Titanar B, Schneider-Kreuznach, Germany). The distance between the specimens and the cameras was set to 1,540 mm, with a field of view of 280 mm × 205 mm, obtaining a pixel size of 0.07 mm.

Before each test, the DIC system was calibrated using a calibration target (Type CP40/200/101296, GOM Aramis, Braunschweig, Germany). This procedure allows to define the physical dimension of the measurement volume, the correction of the distortions due to lenses, and the compensation of the parallax effects (Palanca et al., 2016).

An optimization of the DIC system was performed in order to find the best compromise between the need of reducing the measurement uncertainties, and the desire of obtaining a high measurement spatial resolution. A combination of different values of facet size and grid spacing were investigated for each specimen and the systematic and random errors were computed for each combination of parameters, which are not detailed here for brevity (see Supplementary Material for more details). The following settings were eventually chosen:

• Facet size = 40 pixels;
• Grid spacing = 17 pixels;
• With these settings, a spatial resolution of 2 mm was estimated.

To reduce the amount of data to be stored and analyzed, DIC images were acquired with the following protocol:

• Acquisition of the first 10 cycles;
• Acquisition of the central 10 cycles (from the 45 to the 55 cycles);
• Acquisition of the last 10 cycles.

### 2.6 Analysis of the implant micromotions and strain distribution from the DIC data

**FIGURE 4:**
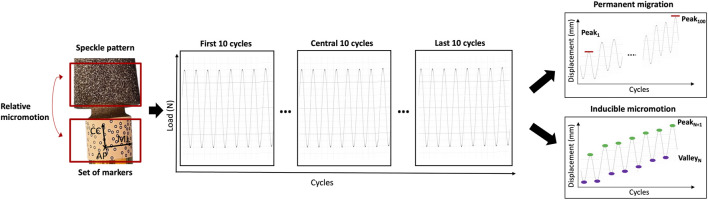
Schematic representation of the analysis of the DIC-measured displacements to compute the permanent migrations and the inducible micromotions. The frames in correspondence of the load peaks and load valleys were extracted. The permanent migrations were computed as the difference between the position of the implant relative to the bone at the last peak and at the first peak (Peak_100_–Peak_1_). The inducible micromotions were computed as the difference between the position of the implant relative to the bone at each load peak and at the corresponding valley (Peak _N+1_–Valley _N_).

Since the DIC allows tracking the motions both of areas with a speckle pattern and of fiducial markers, the spatial micromotions of the prosthesis with respect to the host bone were measured as the displacements (three translations and three rotations) between the distal end of the prosthesis (tracked through the set of markers attached, see paragraph 2.4) and the distal surface of the femur (tracked through the speckle pattern sprayed on the bone, see paragraph 2.4) throughout the test. In particular, the permanent migrations and the inducible micromotions were evaluated. The DIC measurements were post-processed with a dedicated script in Matlab (2021 Edition, MathWorks), which computed (Figure 4):

• The permanent migrations, as the difference between the position of the stem inside the bone at the last and at the first load peak (corresponding to 850 N).
• The inducible micromotion, as the difference between the position of the stem inside the bone at the load peak (850 N) and valley (150 N) of each cycle throughout the test.

**FIGURE 5:**
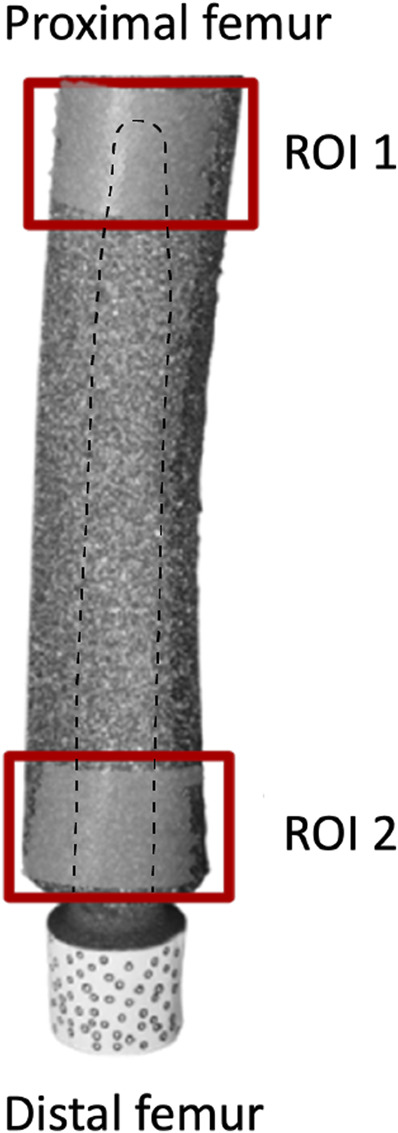
Selection of two regions of interest (ROI). ROI 1 corresponds to the stem tip level, ROI 2 correspond to the osteotomy level. The stem is also reported to better show the position of ROI 1 with respect to the stem tip.

The full-field distribution of the maximum (ε1) and minimum (ε2) principal strains was measured throughout the loading cycles on the entire surface of the femur. The strain distribution at the load peak (850 N) was extracted, taking as a reference frame the initial fully unloaded condition. The investigation focused on two regions of interest (ROI) (Figure 5):

• ROI 1, proximal, was centered on the stem tip and covered the femur from 10 mm proximal to 10 mm distal to the stem tip;
• ROI 2, distal, covered the femur by 20 mm proximal from the osteotomy.

For each ROI, the median value of ε1 and ε2 was computed. The median was chosen as it is a more robust estimator of the average trend, in case of noisy data containing outliers (Montgomery, 2009).

### 2.7 Analysis of the errors of the DIC-measured implant stability

To quantify the systematic and random errors affecting the DIC-measured displacements using a known configuration, a zero-displacement analysis was performed. A pair of subsequent images of the fully unloaded specimen were analyzed with the optimal DIC settings. While in principle this should provide a null displacement, the actual displacement values (in three dimensions) provided by this computation were used as an indicator of the intrinsic measurement uncertainties.

The quality of the experiment in measuring the implant stability was assessed:

• In order to quantify the intra-specimen test repeatability, associated with the variability of the mechanical test, one specimen was tested four times, mounting and dismounting the setup, and reproducing the same loading conditions. The permanent migrations and the inducible micromotions of the four repetitions were compared to quantify the intra-specimen variability affecting the measured implant translations and rotations. It must be noticed that the values reported for the repeatability include both the actual difference among test repetitions, and possible drifts due to specimen conditioning. However, the implants were generally quite stable (see Results below), so the latter component was minimal. Therefore, the values reported are a pessimistic estimate of the actual intra-specimen repeatability.
• In order to quantify the inter-specimen variability, the results of the five specimens, which were tested under identical testing conditions, were analyzed. Specifically, the inducible micromotions and the permanent migrations of the five specimens were compared to quantify the inter-specimen variability affecting the measured implant translations and rotations.

### 2.8 Analysis of the errors of the DIC-measured strain distribution

In order to quantify the systematic and random errors affecting the strain measurements, a zero-strain analysis was performed on subsequent fully unloaded images. In principle, this should provide null strains. The actual strain values (in the different components) provided by this computation were used as an indicator of the intrinsic measurement uncertainties affecting the DIC-computed strains.

To assess the repeatability of the measured strain distribution, the maximum (ε1) and minimum (ε2) principal strains were computed. In detail, the median value of the principal strains was computed at each loading cycle, at 30 Nm, for each of the ROIs:

• In order to quantify the intra-specimen repeatability, the standard deviation of the median principal strains for both ROIs was computed over the four test repetitions of the specimen.
• To quantify the inter-specimen variability, the standard deviation of the median principal strains for both ROIs was computed over the five specimens.

## 3 Results

### 3.1 Errors of the DIC-measured implant stability

The permanent migrations and the inducible micromotions between the prosthesis and the host bone were successfully measured in all the specimens, in all the conditions simulated.

The errors in measuring the implant-bone relative displacements and rotation introduced intrinsically by the DIC were estimated by measuring the roto-translation in a zero-displacement condition: any displacement and rotation value different from zero was accounted for as measurement error. The systematic error was less than 1.5 μm on the displacements, and less than 0.03 degrees on the rotations, in all the directions. The random error on the translations was less than 4.8 μm in all the directions, and on the rotations was less than 0.03 degrees in all the directions.

**FIGURE 6:**
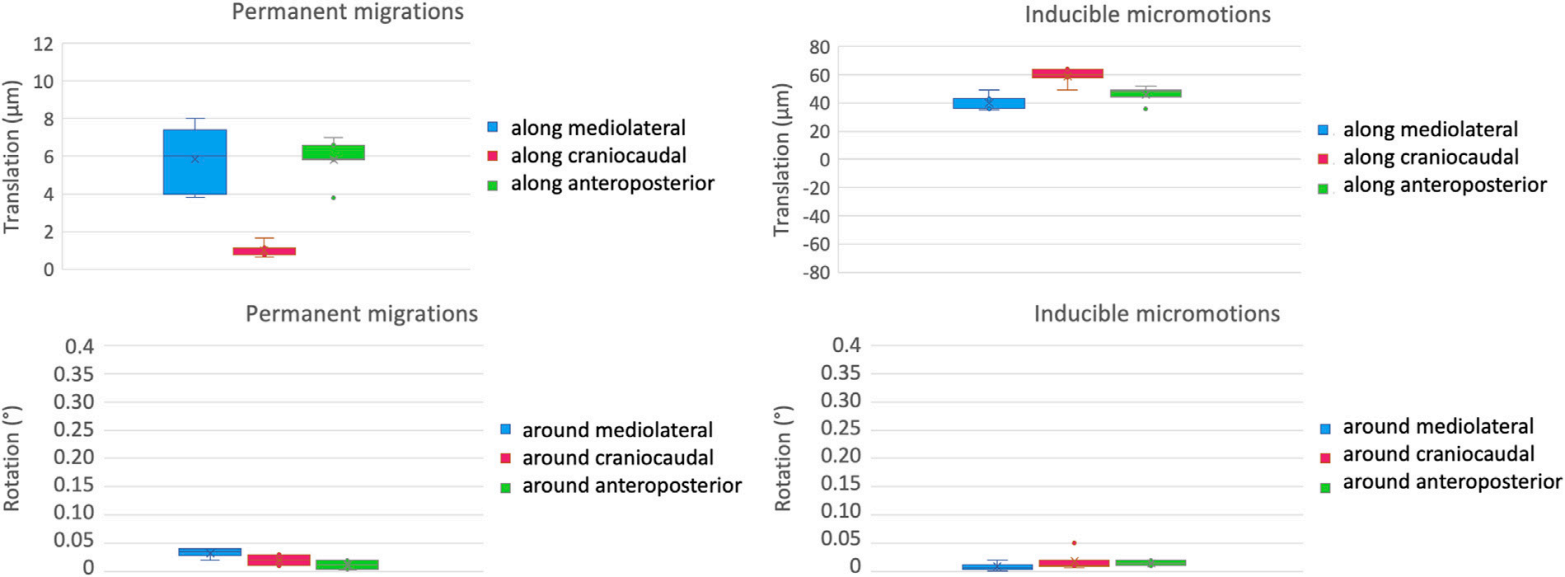
Intra-specimen repeatability: the median and standard deviation of each component of the permanent migrations (left) and inducible micromotions (right) are plotted in terms of translations (top) and rotations (bottom).

The intra-specimen repeatability of the mechanical test was computed as the standard deviation of the four test repetitions of one implant for the micromotions between the prosthesis and the host bone (Figure 6). For the permanent migrations along the mediolateral axis, the craniocaudal axis, and the anteroposterior axis the intra-specimen repeatability was better than 2 μm (translations) and 0.02 degrees (rotations). These errors are smaller than the intrinsic measurement uncertainties. For the inducible micromotions along the directions above, the intra-specimen repeatability was better than 6 μm (translations) and 0.02 degrees (rotations).

**FIGURE 7:**
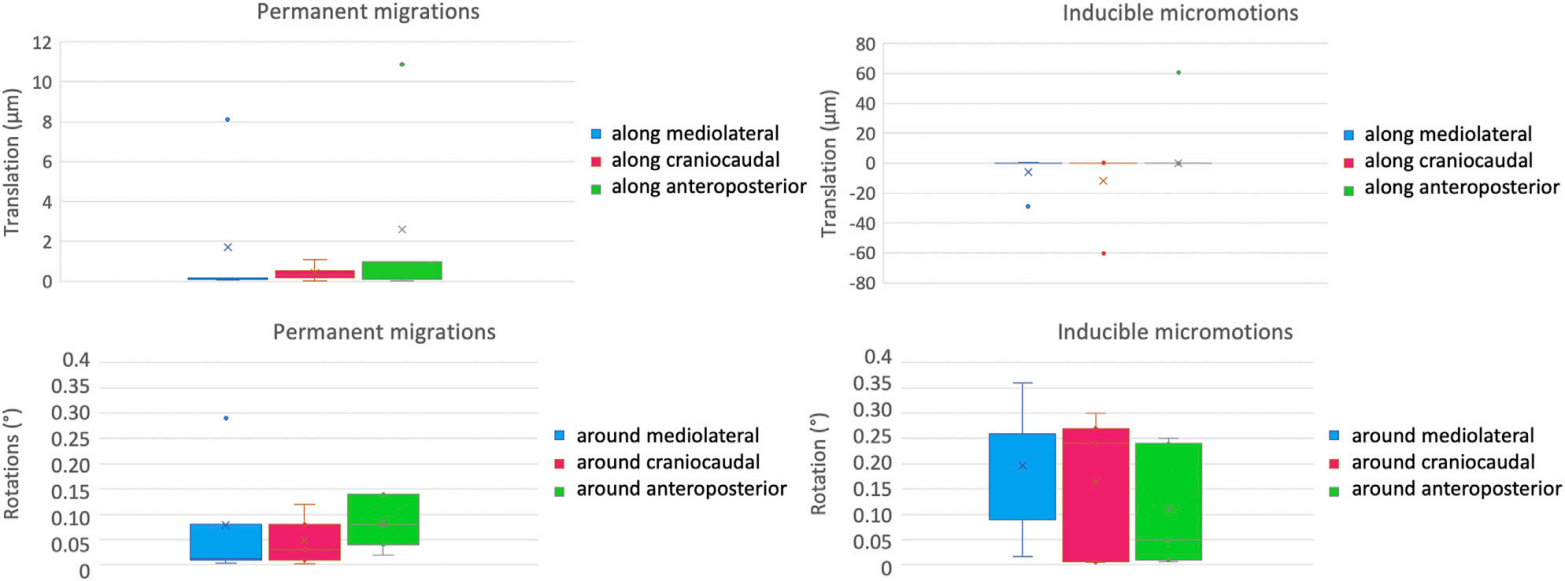
Inter-specimen variability: median and standard deviation of each component of the permanent migrations (left) and inducible micromotions (right) are plotted in terms of translations (top) and rotations (bottom).

The inter-specimen variability was computed as the standard deviation among the five specimens for the micromotions between the prosthesis and the host bone (Figure 7). For the permanent migrations, the inter-specimen variability was smaller than 5 μm for the translations along the mediolateral axis (median = 0.2 μm) and along the anteroposterior axis (median among specimens equal to 1 μm); while was smaller than 1 μm for the translations along the craniocaudal axis (where the median was smaller than 0.1 μm). The inter-specimen variability was 0.12 degrees for the rotations around the mediolateral axis (median 0.01 μm), 0.06 degrees for the rotations around the anteroposterior axis (median 0.08 degrees) and for the rotations around the craniocaudal axis (median 0.03 μm). For the inducible micromotions, the inter-specimen variability was smaller than 13 μm for the translations along the mediolateral axis (where the median was smaller than 0.1 μm); while was 27 μm for the translations along the craniocaudal axis (where the median was smaller than 0.1 μm) and along the anteroposterior axis (median = 0.2 μm). The inter-specimen variability was 0.15 degrees for the rotations around the mediolateral axis (median = 0.26 μm) and for the rotations around the craniocaudal axis (median = 0.24 μm), while was 0.12 degrees for the rotations around the anteroposterior axis (median = 0.05 degrees).

### 3.2 Errors of the DIC-measured strain distribution

The full-field strains distribution was measured successfully in all specimens.

The errors intrinsically introduced by the DIC when measuring the bone strains were estimated by the strains in a zero-strain condition: any strain value different from zero was accounted for as measurement error. The systematic error of the strains on the bone surface in all the repetitions ranged between −11 and +40 microstrain. The random error, instead, ranged between 40 and 66 microstrain.

**FIGURE 8:**
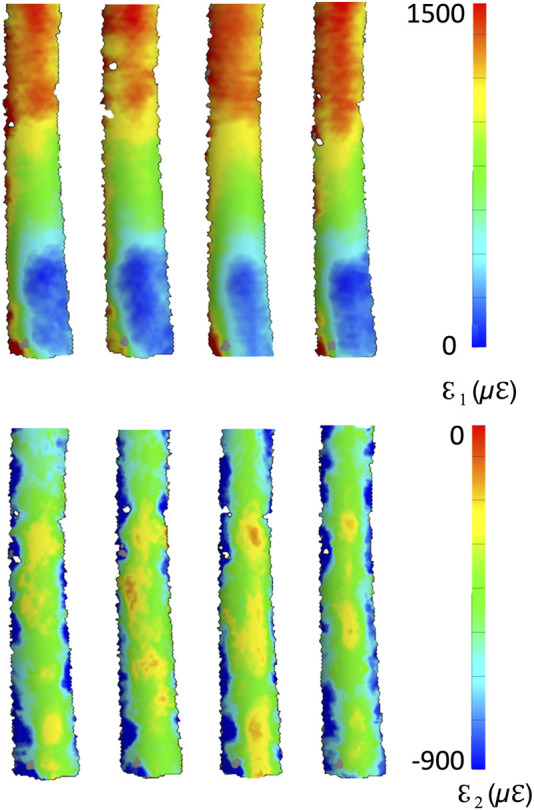
Full-field distribution of the maximum (top) and minimum (bottom) principal strains for four test repetitions over one specimen. The DIC-computed strains at the edges of the correlated region are naturally affected by larger error (Freddi et al., 2015).

**FIGURE 9:**
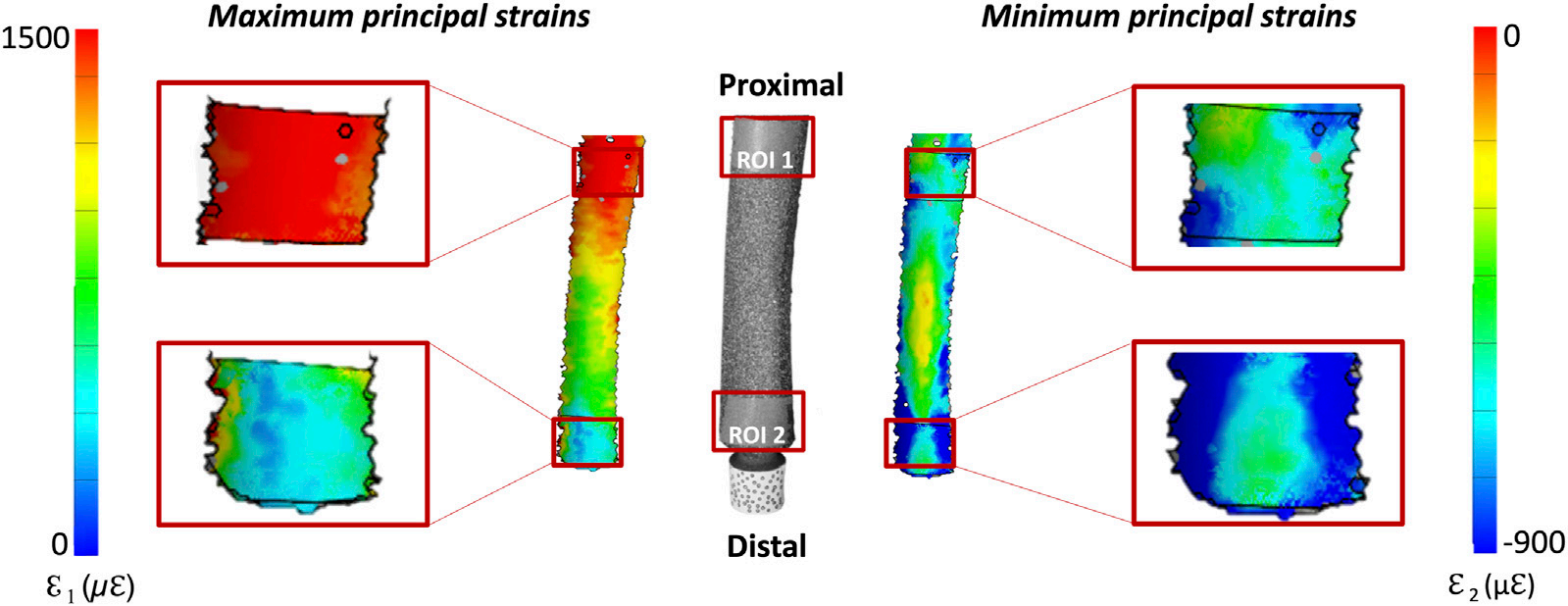
Distribution of the DIC-measured maximum and minimum principal strains: the image in the center shows the actual specimen, as seen by the DIC cameras. The full-field strain distributions, are shown on either side of the bone. The details of the two regions of interest (ROI 1, covering the stem tip level, and ROI 2 corresponding to the osteotomy level) are zoomed on the left and right of the figure.

**FIGURE 10:**
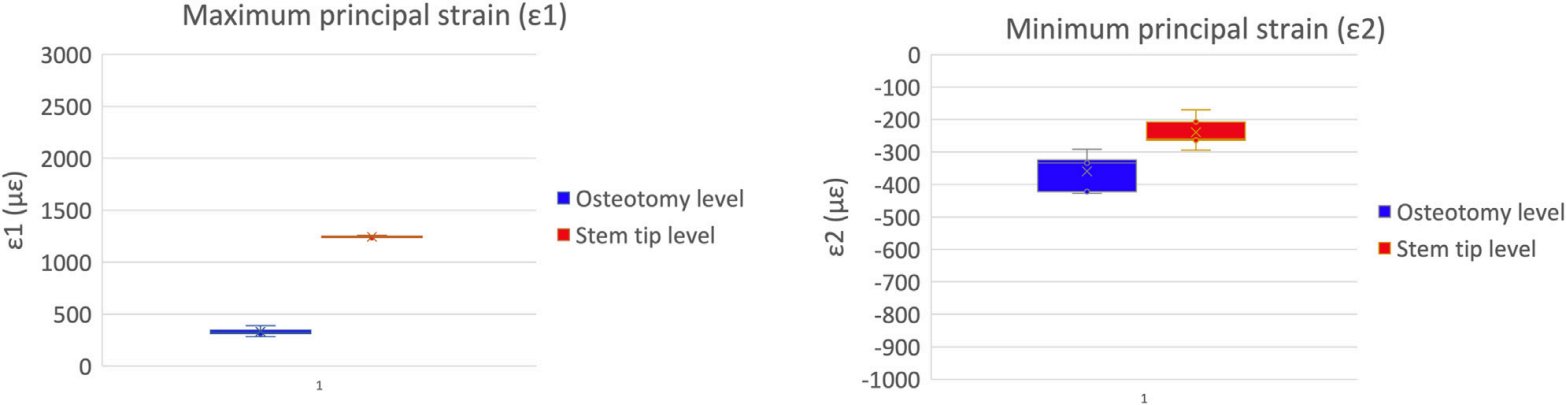
Intra-specimen repeatability: the median and standard deviation are plotted for the maximum (left) and minimum (right) principal strains at the load peak.

**FIGURE 11:**
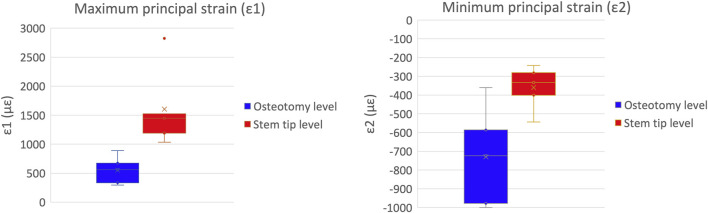
Inter-specimen variability: the median and standard deviation are plotted for the maximum (left) and minimum (right) principal strains at the load peak.

A qualitative inspection of the full-field strain maps showed similar strain pattern (Figure 8). The intra-specimen repeatability and the inter-specimen repeatability were quantified with respect to both ROIs (Figure 9). The intra-specimen repeatability was computed as the standard deviation among the four repetitions for both ROIs (Figure 10). The intra-specimen repeatability for ROI 1 was better than 8 microstrain for the maximum principal strain (ε1), and 59 microstrain for the minimum principal strain (ε2). The repeatability for ROI 2 was better than 35 microstrain for the maximum principal strain (ε1), and 55 microstrain for the minimum principal strain (ε2). The inter-specimen variability was computed as the standard deviation among the five specimens for the strain distribution for both ROIs (Figure 11). The inter-specimen variability for ROI 1 was 700 microstrain for the ε1 (median = 1,604 microstrain) and 270 microstrain for the ε2 (median = −730 microstrain). The variability for the ROI 2 was 245 microstrain for the ε1 (median = 550 microstrain) and 118 microstrain for the ε2 (median = −360 microstrain).

## 4 Discussion

The methods to pre-clinically investigate the possible failure scenarios of osseointegrated transfemoral prostheses are far from consolidated and quite different test conditions, criteria and metrics are reported in the literature (Galteri and Cristofolini, 2023). The main aim of the present study was to devise and validate an *in vitro* mechanical test to reliably assess the primary stability and the load transfer of osseointegrated transfemoral prostheses pre-clinically.

Five implanted femurs were tested simulating the loading condition most frequently experienced during daily life activities, i.e., gait. To detect the primary stability of the implant and the load transfer, cyclic loads were applied to the specimens, simulating the peak load during gait. To the authors’ best knowledge this is the first study in which both the primary stability and the load transfer of an osseointegrated transfemoral prosthesis were measured simultaneously. This was achieved using Digital Image Correlation. The DIC measurements allowed to assess both the permanent migrations and the inducible micromotions between the prosthesis and the host bone in the three components of motion. At the same time, the DIC measurements allowed to assess the full-field strain distribution on the surface of the bone.

To optimize the displacement and strain measurements, the best compromise was sought between measurement uncertainty and spatial resolution. The optimized parameters yielded a spatial resolution better than 2 mm. This is comparable to the grid length of strain gauges typically used in these applications (1–5 mm) (Cristofolini et al., 2009). After optimization, the DIC measurement errors were deemed acceptable for the displacements (below 4.8 μm), the rotations (below 0.03°) and the strains (below 66 microstrain), also in comparison with previous similar studies (Palanca et al., 2016).

The intra-specimen repeatability of the mechanical test was quantified by testing the same specimen four times, mounting and dismounting the setup, and reproducing the same loading conditions. The intra-specimen repeatability of the permanent migrations (both for the translations and rotations) was smaller than the random error of the DIC system, indicating that the loading protocol did not add significant sources of variability. The intra-specimen repeatability of the inducible micromotions was better than 6 μm in all the directions. The intra-specimen repeatability for the rotations of the prosthesis was smaller than the random error of the DIC system in all the directions.

The uncertainties found for the measured micromotions were one order of magnitude smaller than the accepted threshold of inducible micromotions leading to fibrous tissue formation [150 μm (Pilliar et al., 1986)]. This confirms that the sensitivity of this method is sufficient to evaluate the primary stability of an osseointegrated transfemoral prosthesis.

The inter-specimen variability was significantly larger than the intra-specimen repeatability, mainly in relation to the anatomical, bone density and implant variability. The inter-specimen variability for the permanent migrations was better than 5 μm in all the directions, and for the inducible micromotions was better than 30 μm. The inter-specimen variability on the rotation of the prosthesis for both permanent and inducible micromotions was smaller than 0.15 degrees in all the directions. Since no other similar *in vitro* experiment has been published for the osseointegrated transfemoral prosthesis, these values can only be compared against similar tests for uncemented hip stems. The inter-specimen variability in an experiment on hip stems ranged from 50 μm to 150 μm for the permanent migrations and from 5 μm to 30 μm for the inducible micromotions (Cristofolini et al., 2007). The current findings are comparable to previously published results, although the implant, the experiment and the measurement tools were different. The magnitude of the micromotions measured in all the specimens was comparable with the results obtained by Barnes et al. for different prototype prostheses (Barnes et al., 2019). However, they measured micromotions using three LVDTs, and this may lead to errors when estimating motions in space, while DIC allows a full-field 3D displacement analysis.

Moreover, the full-field strain distribution was successfully measured, with an intra-specimen variability on the DIC-measured principal strains lower than 60 microstrain, which is comparable to the random error of the DIC system. These uncertainties should be compared against the strain magnitudes experienced by bone in physiological loading conditions (2000 microstrain (Lanyon, 1980)) and against the failure strains of bone (7,000–10000 microstrain (Bayraktar et al., 2004). The uncertainties deriving from the DIC measurement and from the intra-specimen repeatability were one order of magnitude lower than this range. This confirms that the proposed method is adequate to assess pre-clinically the strain distribution in the host bone, and to detect possible issues with respect to the physiological strain levels and to the risk of fracture.

As expected, the inter-specimen variability for the strains was remarkably larger than the intra-specimen repeatability, due to the natural differences among femurs. For the maximum principal strains (ε1, which was larger in absolute value), the inter-specimen variability was 700 microstrain in the most strained region (ROI 1), and 245 microstrain in the less stressed region (ROI 2). For the minimum principal strains (ε2), the inter-specimen variability was 270 microstrain in the most strained region (ROI 1), and 118 microstrain in ROI 2.

The magnitude of the principal maximum strains measured on the bone surface were within the physiological range of strain for cortical bone in the ROI 1 and decreased towards the distal region (ROI 2) to values below physiological levels of bone (Lanyon, 1980). These strain distributions obtained are comparable to those reported in *in vitro* studies using the strain gauges on selected points (Cristofolini and Toni, 1998; Tomaszewski et al., 2013; Ahmed et al., 2020) and in *in silico* simulations (Tomaszewski et al., 2012; Ahmed et al., 2020).

The developed methodology had some limitations. The main limitation relates to the fact that the proposed experiment can only simulate the early post-operative period, as it is not possible to simulate bone ingrowth and remodeling on *ex vivo* specimens. This is indeed common to all similar *in vitro* studies (e.g., (Østbyhaug et al., 2010; Barnes et al., 2019)). However, the information provided is crucial, as excessive micromotions in the early post-operative period can interfere with the process of osseointegration and can affect the long-term stability. Moreover, this method has been developed for commercially available standard osseointegrated transfemoral prostheses, and adjustments might be necessary if the mechanical test will be carried out on totally different devices.

Indeed, part of the proposed experimental protocol can be adapted to test pre-clinically different osseointegrated devices (e.g., trans-tibial, trans-humeral prostheses). In particular, the experimental protocols to measure the implant micromotions and the full-field strain distribution, and the method for analyzing them could easily be transferred to other types of amputations. Conversely, the direction and the magnitude of the loads are specifically devised for the osseointegrated transfemoral prostheses, and should be re-defined if a different bone segment is to be investigated.

## 5 Conclusion

In conclusion, this work showed that it is possible to simultaneously measure the primary stability and the load transfer of an osseointegrated transfemoral prosthesis in a reliable way using a combination of mechanical testing and DIC. In particular, this study has shown that it is possible to measure the permanent migrations and inducible micromotions (in terms of rotations and translation) and, simultaneously on the same specimen, to measure the full-field strain distribution on the bone surface. The measurement uncertainties (intrinsic measurement errors and intra-specimen repeatability) were small enough to distinguish between stable and unstable implants, and to study the full-field strain distribution on the bone, thus allowing to detect problems pre-clinically.

This method can be applied to different type of osseointegrated transfemoral prostheses. Furthermore, this method can be used synergically with *in silico* models, in order to better predict the post-operative complications of osseointegrated transfemoral prostheses.

## Data Availability

The original contributions presented in the study are included in the article/Supplementary Material, further inquiries can be directed to the corresponding author.
