# Supplementary material for: Reliable in vitro method for the evaluation of the primary stability and load transfer of transfemoral prostheses for osseointegrated implantation
Source: Front Bioeng Biotechnol. 2024 Mar 21;12:1360208. doi: 10.3389/fbioe.2024.1360208 (PMC10991734; doi:10.3389/fbioe.2024.1360208)
Supplement: Supplementary file 1 [file DataSheet1.pdf]

Supplementary Material #1

Additional methodological details and results

Appendix to the paper

Reliable *in vitro* method for the evaluation of the primary stability and load transfer of transfemoral prostheses for osseointegrated implantation

1-Distribution of the speckle pattern

The distribution of the speckle pattern and the dot size were estimated with a dedicated script in Matlab (2021 Edition, MathWorks), which computed:

- The dimension of the dot size of the speckle pattern in pixels;
- The dimension of the dot size of the speckle pattern in mm.

With these parameters the median dot size of the speckle pattern was 3.5 pixels (Figure S1 left) and 0.25 millimeters (Figure S1 right).

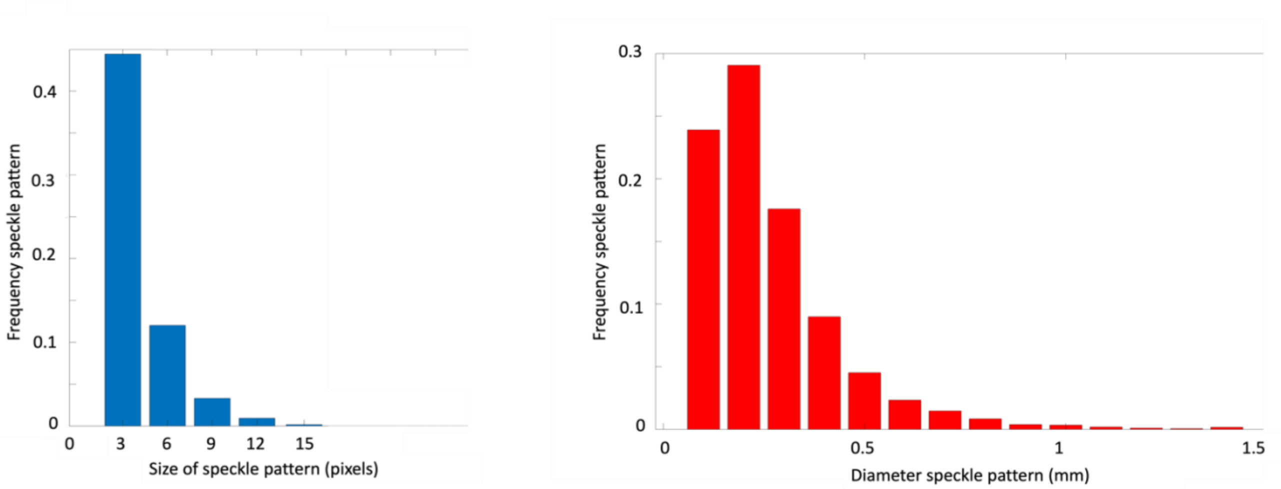

Figure S1: Histograms of the distribution of the speckle pattern sprayed on the surface of the specimens reported in pixels (left) and in millimeters (right).

2- Optimization of the DIC parameters

The software parameters (facet size, grid spacing) were investigated in order to find the best compromise between the need of reducing the measurement uncertainties, and the desire of obtaining a high measurement spatial resolution (Palanca et al., 2015).
A pair of subsequent images of the unloaded specimen were analyzed by changing the parameters of the DIC. The parameters investigated in this work are shown in Table 1.

Table 1: Selection of the software parameters. In the first column are reported the different facet size (FS), while in the second column are reported the point distance (PD).

Facet size(pixels)	Point distance (pixels)
20	8
27	13
30	14
37	15
40	17
47	22
57	26
67	33
77	38

For each combination of the parameters, the strains along the craniocaudal direction were computed in a zero-strain condition. While in principle this should be provide a null strain, the actual strain values provided by this computation were accounted as an error. In particular, the systematic error of each combination was evaluated as the medians, while the random error of each combination was evaluated as the standard deviation.

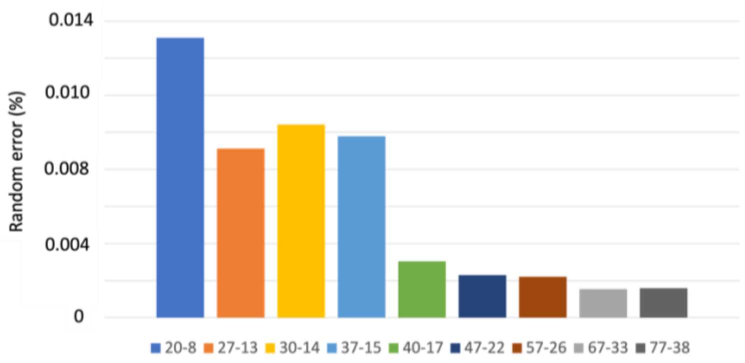

Figure S2: Histogram of the standard deviation of the ε_x for each combination of FS and PD.

The analysis showed that increasing the facet size and the point distance the noise decreased. However, this is associated with a worse resolution (Freddi et al., 2015; Palanca et al., 2015). By limiting the resolution on a limited range needed for this study (at least 2 mm), it was possible select the best software parameters for the specific test and the specific condition:

- Facet size = 40 pixels;
- Grid spacing= 17 pixels;
